# Closing the Gap in Osteoporosis Risk Assessment for Smokers and High-Risk Alcohol Users in a General Practitioner (GP) Practice

**DOI:** 10.7759/cureus.95547

**Published:** 2025-10-27

**Authors:** Basak Selin Kara, Nidhi Sehgal

**Affiliations:** 1 General Practice, Health Education East of England, Cambridge, GBR; 2 General Practice, Milton Surgery, Cambridge, GBR

**Keywords:** dxa, fragility fracture, frax, osteoporosis, primary prevention

## Abstract

Objective

This quality improvement project (QIP) aimed to enhance the identification, management, and documentation of osteoporosis risk among patients aged 50 and above who are both smokers and high-risk alcohol consumers (>14 units/week) in a GP practice.

Methods

The project was conducted over three cycles using the Plan-Do-Study-Act (PDSA) framework. Eligible patients were identified via systematic electronic health record (SystmOne) searches. Interventions included systematic FRAX (Fracture Risk Assessment Tool) scoring for identified high-risk patients, patient contact through text or telephone for those needing further assessment, involvement of practice pharmacist and risk assessment follow-ups, and embedding a FRAX electronic prompt into routine checks at the end. Outcomes measured were FRAX documentation rates, recorded dual-energy X-ray absorptiometry (DXA) result rates, and documentation of appropriate management of DXA results. We assessed statistical significance using Fisher’s exact test.

Results

A total of 49 patients who smoked and consumed alcohol at high-risk levels met the inclusion criteria. At baseline, only one out of 49 eligible patients (2.05%) had a recorded FRAX score. Following three PDSA cycles, FRAX documentation increased to 50 out of 50 patients (100%) (p<0.001), with one additional patient meeting the inclusion criteria during the course of the project. Of 22 patients requiring DXA, only two (9.09%) had scans at baseline, which increased to 11 (50%) following interventions (p=0.0068). Two (22.2%) of nine patients who needed and had a DXA scan for assessment after interventions had osteoporosis, five (55.6%) osteopenia, and two (22.2%) normal bone density. Interventions were initiated following the National Osteoporosis Guideline Group (NOGG) guidelines.

Conclusions

This quality improvement initiative showed that systematic changes can improve adherence to osteoporosis prevention measures within a primary care setting. Electronic health record searches and involvement of a multidisciplinary team enabled improvements in both the identification of high-risk patients and their clinical management. However, the single-site setting, limited cohort size, and short follow-up period, as well as dependency on accurate electronic documentation, restrict generalisability. Targeted QI interventions can cause substantial improvements in fragility fracture risk assessment and DXA scanning and preventive measures in high-risk groups within primary care. Embedding prompts in electronic health systems and involving multidisciplinary team members are practical strategies to increase fracture prevention. Future expansion to other risk groups and evaluation of long-term outcomes will be essential to facilitate further improvement in the prevention of fragility fractures.

## Introduction

The World Health Organisation (WHO) defines osteoporosis as a systemic skeletal disorder that results in low bone mass, causing an increase in bone fragility and risk of fracture [1]. In the United Kingdom, an estimated 3.775 million people are living with osteoporosis, affecting around 21.9% of women and 6.7% of men aged 50 years and older [2,3]. WHO has defined osteoporosis as a bone marrow density (BMD) value 2.5 standard deviation (SD) or more below the young adult mean (T-score ≤ −2.5), while osteopenia is defined as a reduced BMD with a T-score between −1.0 and −2.5 [1]. BMD is most commonly measured by utilising a dual-energy X-ray absorptiometry (DXA) scan to assess fracture risk [1]. 

Osteoporosis causes fragility fractures mostly in the bones of the hip, spine, and wrist. These fractures are found to be associated with significant morbidity and mortality [2,3]. It is estimated that 549,000 new fragility fractures occur each year in the United Kingdom, including approximately 105,000 hip fractures and 86,000 vertebral fractures [4]. Despite the high prevalence of osteoporosis and fragility fractures, the UK ranked 23rd among EU+2 countries in 2019 in terms of access to DXA scanning [5]. Several risk factors have been identified for the development of osteoporosis and fragility fractures. Kanis et al. demonstrated that current smoking increases fracture risk by 25% (relative risk: 1.25, 95% CI: 1.15-1.36) [6]. Similarly, regularly consuming two or more standard drinks of alcohol per day is associated with a 1.63-fold increase in fragility fracture risk [7]. Both smoking and high-risk alcohol use are also associated with a higher prevalence of chronic conditions such as diabetes mellitus and chronic liver disease that increase the risk of secondary osteoporosis and fracture [8,9].

Applying primary prevention measures in the population at high risk is crucial to prevent fragility fractures and osteoporosis. Both the National Institute for Health and Care Excellence (NICE) and the National Osteoporosis Guideline Group (NOGG) recommend fracture risk assessment in adults aged ≥50 years with high-risk factors, including smoking, high-risk alcohol intake (>14 units per week). For risk assessment, FRAX (Fracture Risk Assessment Tool) is recommended to be utilised to guide decisions on bone density testing with DXA and treatment [10,11]. Despite these recommendations, the identification of high-risk individuals and the management of osteoporosis in primary care are often given lower priority compared to other long-term conditions within European healthcare systems [7]. 

This project aimed to improve the identification, management, and recording of fragility fracture risk in patients aged 50 and over who are both smokers and high-risk alcohol users (>14 units/week) in a GP practice. The objectives included increasing FRAX assessment and recording, ensuring appropriate DXA referrals when indicated, and optimising the management and follow-up of results.

## Materials and methods

Setting

This quality improvement project (QIP) was undertaken at a UK general practice with approximately 5,000 registered patients in a rural setting. The practice team included general practice consultants and a registrar, nurses, pharmacists, and administrative staff, who supported implementing workflow changes aimed at enhancing primary prevention of fragility fractures. The project was approved internally as a quality improvement initiative.

Population and inclusion criteria

Patients aged ≥50 years who were current smokers and reported high‑risk alcohol consumption (>14 units/week) were identified as the target population using coded information on the practice’s electronic medical record system. This population was selected in accordance with NICE Clinical Knowledge Summaries and NOGG recommendations on conducting a risk assessment for patients at high risk of fragility fractures. NOGG and NICE guidelines emphasise smoking and high-risk alcohol use as modifiable risk factors for fragility fractures and recommend that the population above 50 years of age be assessed if they are at high risk. Patients with a documented history of prior fragility fractures on SystemOne were excluded to avoid misclassification. Patients meeting these criteria were identified through a comprehensive search on electronic health records via SystmOne on February 5, 2025. 

Baseline measurement

We recorded the number of eligible patients, the number of patients with documented FRAX scores, and existing DXA scan records. At baseline, 49 patients met the inclusion criteria; one (2.05%) had a documented FRAX score in the system records.

Standards and targets

Based on NICE Clinical Knowledge Summaries and NOGG recommendations, standards were set as follows: (1) 100% of eligible patients should have a recorded FRAX score; (2) 100% of patients at intermediate or high risk on FRAX assessment should be referred for DXA; and (3) 100% of DXA results should be followed and management should be documented. Appropriate management of osteoporosis is defined as antiresorptive therapy, assessment of calcium and vitamin D intake with replacement if needed, and provision of lifestyle advice, while appropriate management of osteoporosis is set as assessment of calcium and vitamin D intake with replacement if indicated, and provision of lifestyle advice as per NOGG and NICE guidelines.

Interventions

**Figure 1:**
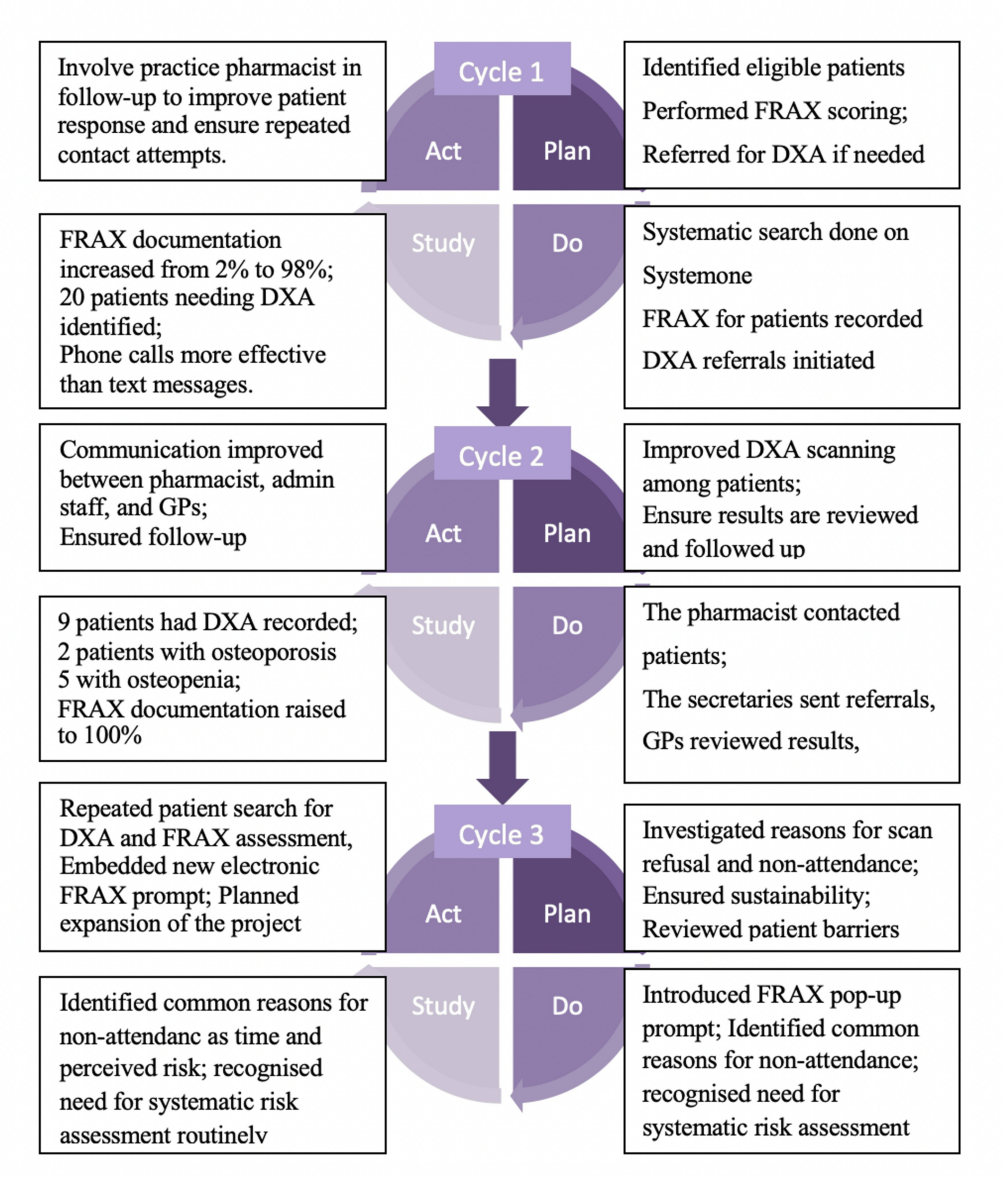
Summary of PDSA cycles FRAX: Fracture Risk Assessment Tool; DXA: dual-energy X-ray absorptiometry

In this project, we utilised the Plan-Do-Study-Act (PDSA) format. During PDSA Cycle 1, FRAX scores were recorded for all eligible patients, and, based on these scores, referrals for DXA scans were planned for completion. In PDSA Cycle 2, a practice pharmacist was involved to support and improve DXA referrals, tracking scan results, and arranging follow-ups. In PDSA Cycle 3, we explored reasons for failure in completion of all assessments and embedded a pop‑up FRAX prompt within SystmOne for new patient and chronic disease check templates to sustain changes. Interventions in each cycle are illustrated in Figure 1.

Data collection and analysis

Data were collected from the practice’s electronic medical record system, SystmOne. A targeted search on Systemone identified patients aged 50 years and older who are smokers and high-risk alcohol consumers (>14 units/week). Outcomes were measured by identifying the proportion of eligible patients who underwent FRAX assessment, the number of patients with a recording of DXA scan results. Baseline data were collected before the intervention. Outcomes were assessed by auditing these records after the completion of each PDSA cycle. Subsequent data were recorded after each PDSA cycle to track improvements in FRAX documentation and DXA referral and follow-up recordings. Patient engagement and barriers to completing assessments were also noted. All data were collated and analysed utilising Microsoft Excel. Results were compared against baseline performance. Statistical significance was analyzed using Fisher’s exact test. A p-value below 0.05 was considered statistically significant.

Ethical considerations

This QIP does not meet the criteria for human subject research based on UKHRA (UK Health Research Authority) criteria since it does not involve randomisation, does not change standard patient care, and its results are not generalisable. Therefore, ethical approval was not required. The project was locally registered as a Quality Improvement Project. The publication manuscript adheres to SQUIRE (Standards for Quality Improvement Reporting Excellence) V2.0 guidelines [12].

## Results

Of the 5,000 registered patients at the practice, 49 met the criteria for inclusion in the quality improvement project. At baseline audit, among 49 eligible patients (≥50 years, smokers with high‑risk alcohol consumption), only one (2.0%) had a recorded FRAX score, while 48 (98.0%) had no FRAX score documented. After PDSA Cycle 1, the percentage of patients with a recorded FRAX rose to 49/50 (98.0%), including one additional patient who met the inclusion criteria during the timeframe of the first cycle. The patient who met the criteria during the timeframe of the first cycle was included in all subsequent analyses beginning with the outcomes of Cycle 1. Among the 49 patients with a recorded FRAX score at the end of the first cycle, 22/49 (44.9%) were at high or intermediate risk for osteoporosis based on FRAX scoring; thus, they required DXA scanning to further identify their risk. Two (9.1%) of those 22 patients who required DXA scanning already had a DXA result recorded at baseline.

**Figure 2:**
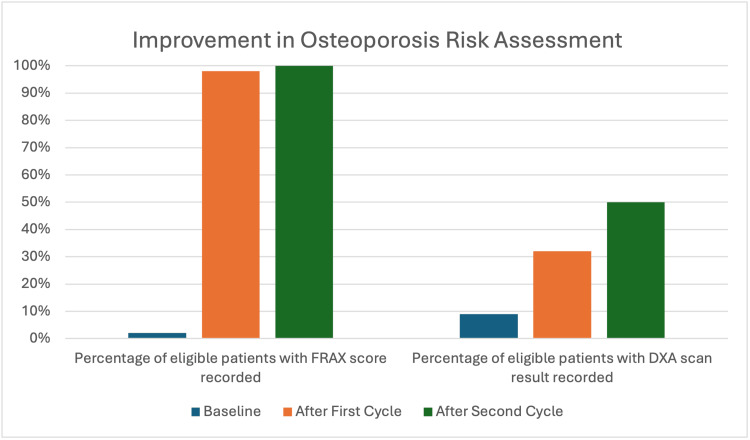
Improvement in osteoporosis risk assessment recordings FRAX: Fracture Risk Assessment Tool; DXA: dual-energy X-ray absorptiometry

At the end of the second PDSA cycle, 50 patients were meeting the inclusion criteria, and FRAX recording in the system reached 50/50 (100%) in the eligible group, which demonstrated a statistically significant improvement in FRAX assessment in the risk group at the end of the project (p<0.001). Despite 22 patients being eligible, only 2/22 (9.1%) had a DXA result recorded at baseline. After the first two cycles with the pharmacist's involvement, nine new patients out of 22 in need had DXA scan results recorded. This showed a significant increase in the proportion of eligible patients who had DXA recorded from 2/22 (9.1%) to 11/22 (50%) (p=0.0068). These results demonstrated a marked improvement in appropriate risk assessment in GP surgery in the defined risk group compared to baseline. The improvements in FRAX score assessment and recording, as well as DXA scan assessment and recording, are shown in Figure 2. However, patient engagement in DXA scan assessment presented challenges, as some patients declined the scan due to time constraints and a perceived low risk of the condition.

DXA results were reviewed at the end of the second and third PDSA cycles. Among those nine patients who had risk assessment at the end of the third cycle, two (22.2%) patients were diagnosed with osteoporosis, five (56.6%) patients with osteopenia, and two (22.2%) patients had normal bone density.

Patients with osteoporosis whose fracture risk exceeded the NOGG treatment threshold were started on oral alendronate as antiresorptive therapy and advised on lifestyle measures - regular exercise, adequate calcium/vitamin D intake, smoking cessation, alcohol reduction, and a balanced diet - according to NOGG guidance. They were provided with calcium supplementation when Ca intake was found to be insufficient (less than 700 mg daily) and vitamin D supplements to ensure at least 800 IU/day intake during GP assessment as per NOGG guidelines. Patients with osteopenia were also provided with calcium and vitamin D supplementation based on their calcium and vitamin D intake status, assessed by the contacting clinician, and they were provided with lifestyle advice. Patients with normal DXA results also received lifestyle guidance focusing on smoking cessation and reducing alcohol consumption. These findings demonstrate that the intervention not only improved assessment processes but also led to appropriate clinical management of patients.

In the third PDSA cycle, a FRAX pop-up prompt was created and introduced into the electronic health record system to be utilised during both new patient checks and chronic disease reviews to ensure sustainability of the improvement in fragility fracture risk assessment in GP practice. Long-term effectiveness and sustainability of FRAX pop-ups will be monitored with future audits assessing compliance with FRAX assessment advice for patients with a high risk of fragility fractures. The practice also agreed on a plan to expand the project gradually to include other high-risk groups for osteoporosis.

Overall, FRAX documentation among the identified risk group rose from 1/49 (2.0%) to 50/50 (100%) at the end of the PDSA cycles. The proportion of eligible patients who had appropriate DXA assessment and recording increased significantly from two out of 22 (9.1%) at baseline to 11 out of 22 (50%) after three PDSA cycles.

## Discussion

Fragility fractures have emerged as a major public health concern globally, particularly with the aging population, as they are associated with substantial morbidity, mortality, and healthcare costs. Within the European Union, approximately 4.3 million fragility fractures are estimated to occur each year [13]. The ScoreCard for OsteoPorosis in Europe (SCOPE) states that osteoporosis management across Europe is not standardised, which results in an important gap in the treatment of osteoporosis and the prevention of fragility fractures [5]. Similarly, in the UK, there were more than 527,000 new fragility fractures documented in 2019 [3]. Although there are clear guidelines highlighting osteoporosis management from national bodies such as NOGG and NICE that promote fracture risk assessment and management, implementation of these recommendations in daily practice remains inconsistent [10,11].

A retrospective audit revealed that only 16% of patients admitted to a hospital for fragility fractures were prescribed bisphosphonate during their hospital stay [14]. Furthermore, roughly 87% patients with fragility fractures did not receive antiresorptive therapy from their general practitioners [14]. A multi-centre audit revealed similar results, as only 47% of patients were prescribed bone-sparing treatment after fragility fractures [15]. This proportion has increased to 84% after the allocation of a community-based fracture liaison nurse [15]. The same study revealed that risk factors for fragility fractures, such as smoking status and high BMI, were under-recorded before this intervention [15].

Our QI project demonstrated outcomes consistent with those reported in existing literature. Initially, only 2% of patients in our GP practice who are smokers and high-alcohol users aged ≥50 and therefore at high risk of fragility fractures had a FRAX assessment recorded. Following the implementation of PDSA cycles, FRAX documentation in the defined risk group improved to 50/50 (100%). As a result, the DXA scan rate in the population who needed a scan for further assessment of fragility fracture risk has increased significantly. This result showed that systematic and focused changes in practice can effectively address gaps in primary prevention of fragility fractures, aligning with the NOGG guidelines that recommend osteoporosis risk assessment in the population aged ≥50 with risk factors [11].

Utilising electronic health records and allocating jobs to multidisciplinary teams are strategies that improve screenings and treatment in healthcare settings [7,16]. A service evaluation project showed that introducing a new computer-based system improved the osteoporosis referrals [16], while a UK-based primary care QIP demonstrated improvement in osteoporosis guideline adherence by involving nurses in fragility fracture management [7]. In our project, integrating a FRAX pop-up into the online record system for new patient and chronic disease checks represented a cost-effective use of the electronic health system to sustain improvements in fracture risk assessment. Moreover, involving the practice pharmacist in risk assessment and follow-up facilitated increased DXA referrals and appropriate management. Similarly, a previous study showed that community pharmacist-initiated screening was an effective way of improving osteoporosis risk assessment in the local population [17]. The sustainability of improvements in fragility fracture risk assessments was planned to be evaluated and supported through annual audits. Previous projects have demonstrated that regular audits and targeted interventions promote long-term enhancement in osteoporosis management and risk assessment practices [18].

Our project has several limitations. This quality improvement project was conducted in a single rural general practice with a relatively small registered patient population. Therefore, the results may not be generalised to larger cohorts. The risk group chosen in the project was targeted to facilitate the appropriate use of resources, and the risk group represented only one subset of high-risk individuals. The patients were identified from the electronic health recording system; therefore, the identification of high-risk patients depended on accurate documentation in the GP practice. The barriers for patient engagement were not assessed with a standard method, although these were noted when patients reported during the encounters. Patient-reported outcomes, such as satisfaction with service, were not explored in the project. A standard survey including questions on barriers and the positive impact of the assessment on patients' satisfaction could be added in future QI projects. Extending the project to cover other risk groups, such as corticosteroid users, is planned for future projects. Additionally, the intervention was re-assessed after three months; thus, to assess long-term sustainability, another cycle can be conducted, although the project demonstrated significant improvement in the short term.

Despite these limitations, this quality improvement project demonstrated several significant strengths. Firstly, the implementation of quality improvement measures resulted in a rapid and measurable improvement in the appropriate primary prevention of osteoporosis in the target risk group in three months. The multidisciplinary team, including GPs, pharmacists, the administrative team, and the project lead, facilitated efficient implementation of the interventions. Embedding a FRAX pop-up prompt into the electronic health record system will provide a sustainable mechanism to maintain risk assessment in routine practice. Furthermore, this project helped raise awareness across the general practice team and enabled discussions on primary prevention of fragility fractures during clinical meetings and audit presentations. These changes represent the efficiency of QI methodologies in enhancing preventive care delivery in primary care.

## Conclusions

This QIP demonstrated that implementing targeted interventions can substantially improve adherence to osteoporosis risk assessment and management guidelines in primary care. By involving the practice team and conducting systematic searches of electronic health records, improvements in the primary prevention of fragility fractures were achieved among smokers and heavy alcohol users aged over 50 years. At the end of the project, FRAX documentation among the target GP practice population improved from 2% at baseline to 100% following two PDSA cycles. DXA completion among patients who needed DXA for the appropriate assessment of fragility fracture risk rose from 9% to 50% at the end of the third cycle. Treatment and lifestyle advice were provided to patients diagnosed with osteoporosis or osteopenia following NICE and NOGG guidelines. Overall, this QIP demonstrated that simple interventions utilising electronic systems and incorporating allied healthcare professionals can lead to significant improvements in preventive care. Future extension to additional high-risk populations, along with evaluation of long-term outcomes, will be critical in promoting continued improvements in fragility fracture prevention.
